# Rapid ammonia build-up in small individually ventilated mouse cages cannot be overcome by adjusting the amount of bedding

**DOI:** 10.1038/s41684-023-01179-0

**Published:** 2023-05-18

**Authors:** Mahmud A. Eskandarani, Jann Hau, Otto Kalliokoski

**Affiliations:** Department of Experimental Medicine, Faculty of Health and Medical Sciences, University of Copenhagen, Copenhagen, Denmark

**Keywords:** Translational research, Animal physiology

## Abstract

We sought to investigate if varying levels of bedding had an effect on intra-cage ammonia levels in individually ventilated mouse cages (Euro Standard Types II and III). Employing a routine 2 week cage-changing interval, our goal is to keep ammonia levels under 50 ppm. In smaller cages used for breeding or for housing more than four mice, we measured problematic levels of intra-cage ammonia, and a considerable proportion of these cages had ammonia levels at more than 50 ppm toward the end of the cage-change cycle. These levels were not reduced significantly when the levels of absorbent wood chip bedding was either increased or decreased by 50%. The mice in both cage types II and III were housed at comparable stocking densities, yet ammonia levels in larger cages remained lower. This finding highlights the role of cage volume, as opposed to simply the floor space, in controlling air quality. With the current introduction of newer cage designs that employ an even smaller headspace, our study urges caution. With individually ventilated cages, problems with intra-cage ammonia may go undetected, and we may opt to utilize insufficient cage-changing intervals. Few modern cages have been designed to account for the amounts and types of enrichment that are used (and, in parts of the world, mandated) today, adding to the problems associated with decreasing cage volumes.

## Abstract (web summary)

The authors investigated the influence of housing density and bedding volume on ammonia build-up in two individually ventilated mouse cages models. While the amount of bedding had no influence on ammonia levels, smaller Type II individually ventilated cages accumulated higher ammonia levels than larger Type III individually ventilated cages, even at similar housing densities.

## Main

Housing laboratory rodents in individually ventilated cage (IVC) systems ostensibly improves the environment of both animals and their keepers. Compared with traditional open-cage housing, IVCs reduce the risk of infections spreading rapidly in animal colonies, while allowing for better control of the microclimate in the cages with respect to temperature, humidity and air quality^1,2^. For the animal caretakers, isolating the air from the cages reduces the spread of allergens, removes odor and introduces a physical barrier shielding them from potentially toxic compounds with which animals may be dosed^3–5^. Air quality in cages is easily maintained through a high (active) ventilation rate^6^. All the while, room-level ventilation—managing a substantially larger volume of air—can be reduced^7^, thereby reducing power costs. Consequently, compared with open cages, IVC systems offer other benefits, such as reducing facility management costs, given that heating, ventilation and air conditioning systems are the biggest operating expense in animal facilities outside of personnel^8,9^. This is compounded with a move toward an extended, 2-week, cage-change cycle driven by improvements in intra-cage environment^10^, leading to even more savings.

Importantly, IVC systems have been shown to be superior to, for example, filter top cages, in reducing ammonia (NH_3_) build-up in cages^11–13^. Bacteria shed by the occupants of the cages can metabolize urea (CO(NH_2_)_2_) in urine, forming ammonia (NH_3_)^14,15^. Over time, this process creates a compromised environment^16,17^. Ammonia is a weak base, corrosive to organic material, which will spread in aerosolized form. Mammalian species have evolved to detect even minute concentrations of NH_3_ in air as a noxious odor. Most people can detect concentrations of NH_3_ well below 10 ppm (ref. ^18^). As airborne droplets of NH_3_ react with the mucous membranes in the upper airways and around the eyes, ammonia induces discomfort^19,20^. Over time, corrosive damage will manifest, having an adverse effect on human and animal health^21–24^. With exposures to high concentrations of NH_3_, the body will mount a response—such as coughing and changed breathing rate—in an attempt to prevent damage to the lungs^25^.

Whereas most countries will have occupational exposure limits restricting worker exposure to ammonia (often set at 20–25 ppm averaged over a workday, with higher levels allowed for short periods^26–28^), there are currently no agreed-upon limits for animals, such as laboratory mice. Given that NH_3_ build-up in cages with soiled bedding is a historical management issue in laboratory animal facilities, numerous studies into the effects of NH_3_ exposure in mice have been carried out^29^. Whereas there is conflicting evidence with respect to the effects of low levels of intra-cage NH_3_ on rodent health, there are no reports—at least that we are aware of—that have not found adverse effects (mainly lesions of the upper airways/nasal passage) when housing rodents in individually ventilated environments where NH_3_ levels exceeded 50 ppm (refs. ^22,23,30–32^). This value, consequently, has been suggested by Silverman et al.^33^ as the level at which cages should be changed at the latest.

Compared with traditional open cages, animal caretakers are less likely to detect ammonia build-up with IVC systems, in particular when the IVCs are operated under negative pressure and the rack exhaust is ducted directly from the animal room. Only when the cages are removed from the racks and opened—often in a process ventilated area—can the smell of ammonia be detected. But even then, the relatively small volume of air inside the cage will equilibrate with the considerably larger volume of air in the cabinet or room. We suspect that animal caretakers are, thus, likely to greatly underestimate ammonia build-up in IVCs.

Following routine sampling of cages at our facility, we were made aware of cages reaching ammonia levels exceeding 50 ppm, prompting thorough investigation. In this Article, we investigated the influence of cage occupancy and bedding volume on the ammonia build-up in two commonly used IVC models. The aim was to determine which cages were at risk of developing ammonia levels exceeding 50 ppm in a regular cage change cycle, and to determine whether this could be counteracted through more appropriate use of absorbent bedding.

## Results

**Fig. 1:**
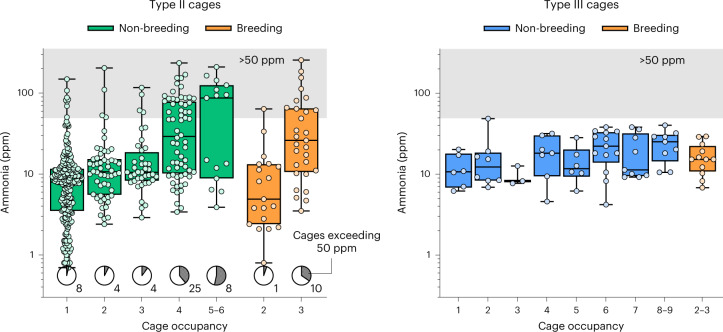
Sampling representative cages. Intra-cage ammonia is shown arranged by cage occupancy for small (Type II; left) and larger (Type III; right) IVCs. Measurements were obtained 10–14 days after cage change through random sampling. Note that data are shown on a logarithmic axis. Boxes display medians and the inter-quartile range, whiskers denote full range of the data. The gray area is used to highlight the area of readings exceeding 50 ppm NH_3_. The number and proportion of Type II cages exceeding the 50 ppm threshold is indicated at the bottom of the graph. For the exact composition of groups, see Table 1.

**Extended Data Fig. 1:**
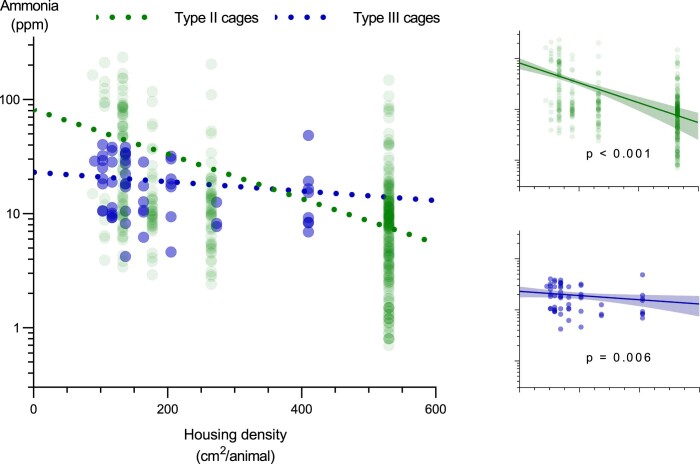
Regression analysis for intra-cage ammonia as a function of cage occupancy. Best-fit regressions are shown for both cage Types (II, in green; and III, in blue) over the same housing density range (data from Experiment 1). Breeding cages have been excluded from the analysis. On the right, the regressions are shown individually, with the 95% confidence interval for the regression lines (with p-values listed for the associated analysis of variance) for a less cluttered view. Note that statistical testing was carried out as linear regression analysis of log-transformed data, whereas the data is here shown in a semi-log plot.

When sampling randomly selected cages (summarized in Table 1) after 10–14 days on the same bedding (Fig. 1), a clear relationship could be found in the smaller Type II cages between the number of mice in a cage and the resulting ammonia levels (regression analysis; *F*_1,401_ = 123, *P* < 1 × 10^−24^; Extended Data Fig. 1). Irrespective of cage occupancy, we found Type II cages with ammonia build-up exceeding 50 ppm (Fig. 1). The problem was, however, particularly prominent in non-breeding cages with more than four mice, and in cages housing breeding trios. By contrast, none of the larger Type III cages were measured at more than 50 ppm on day 14 (Fig. 1). There was also a much weaker relationship between the cage occupancy and the resulting ammonia levels for Type III cages (regression analysis; *F*_1,56_ = 8.11, *P* = 0.006; Extended Data Fig. 1).

**Table 1:** Sampling of representative cages

Mice per cage	Non-breeding									Breeding	
	1	2	3	4	5	6	7	8	9	Pairs	Trios
**Smaller (Type II) cages**											
Number of cages (*n*)	230 M: 205 F: 25	54 M: 35 F: 19	40 M: 25 F: 15	64 M: 18 F: 46	13 M: 7 F: 6	2 M: 0 F: 2	–	–	–	19	29
Floor space per animal (cm^2^)	530	265	177	133	106	88	–	–	–	265	177
**Larger (Type III cages)**											
Number of cages (*n*)	6 M: 5 F: 1	8 M: 3 F: 5	3 M: 1 F: 2	7 M: 0 F: 6 Mix: 1	6 M: 1 F: 3 Mix: 2	13 M: 3 F: 9 Mix: 1	9 M: 4 F: 5	8 M: 0 F: 7 Mix: 1	1 M: 0 F: 1	1	11
Floor space per animal (cm^2^)	820	410	273	205	164	137	117	103	91	410	273

The table lists the number of cages that were sampled, listed by cage occupancy. Some larger cages may have had breeding constellations, but this could not be immediately assessed from a visual inspection of the cage. ‘Mix’ refers to such a cage with both males and females. These are however likely to be litters of sexually immature mice that have been weaned early. M, male; F, female.

**Extended Data Fig. 2:**
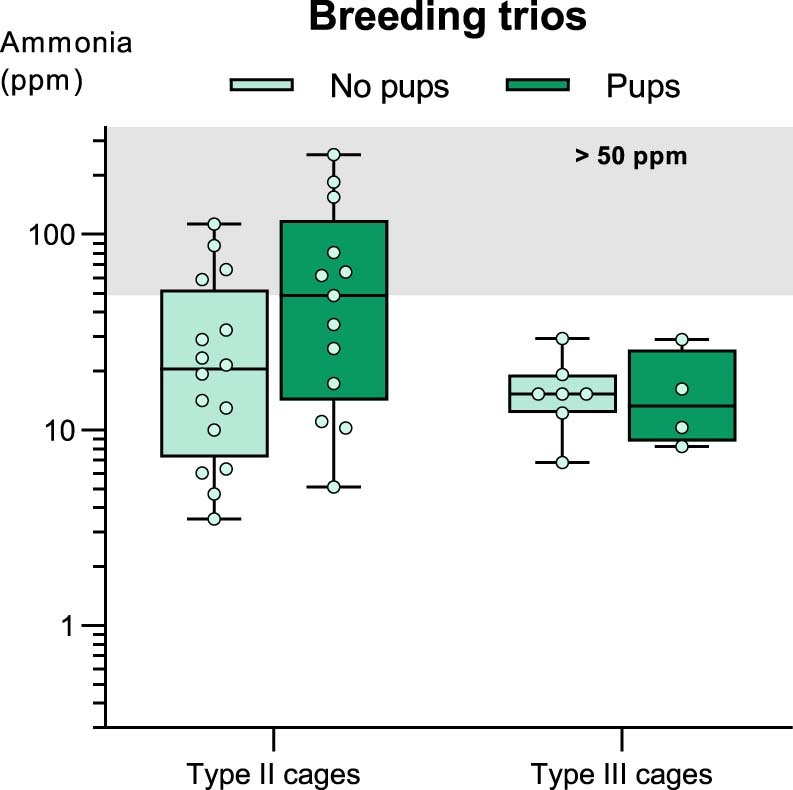
Ammonia levels in breeding cages with and without a litter of pups. The breeding cages housing trios have been divided by whether there was a litter of pups recorded in the cage at the time of sampling or not. Although there appears to be an increase in intra-cage ammonia in the smaller (Type II) cages, this effect could not be statistically substantiated. Boxes display medians and the inter-quartile ranges, whiskers denote full range of the data.

**Extended Data Fig. 3:**
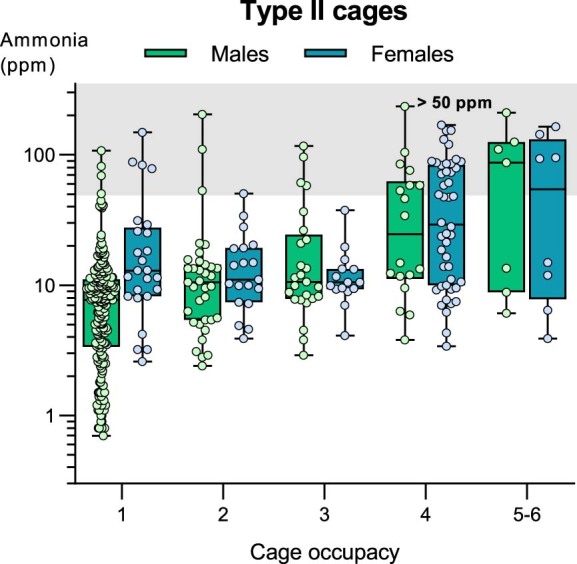
Ammonia levels of type II cages split by sex. The Type II cages in Experiment 1 (Fig. 1) have been split by sex (see Table 1). There are no obvious differences between males and females to suggest that sex has a deciding effect on ammonia build up in these cages. Two-way analysis of variance supports this finding. Boxes display medians and the inter-quartile ranges, whiskers denote full range of the data. The gray area is used to highlight the area of readings exceeding 50 ppm NH_3_.

With such an exploratory design, we have refrained from excessive hypothesis testing; we will however use our data to verify some assertions we had made on the basis of previous observations. Animals housed at comparable densities in the two cage types would be exposed to substantially higher ammonia levels if they were housed in a Type II cage. Comparing animals housed at a density of 140 cm^2^ per animal or lower (that is, denser) revealed a clear difference between cage types (Welch’s *t*-test; *t*_104.2_ = 2.43, *P* < 0.017). Small, Type II, breeding cages were not, overall, more prone to developing high ammonia levels than were non-breeding cages with the same number of mice (two-way analysis of variance (ANOVA); *F*_1,138_ = 0.21, *P* = 0.64). There was however a strong interaction between the type of cage and its occupancy (*F*_1,138_ = 15.0, *P* < 0.001); indeed, whereas there was no clear difference between breeding and non-breeding Type II cages housing pairs, breeding trios had, on average, much greater ammonia levels than did non-breeding cages of three mice. We could not attribute this increased ammonia build-up in trio breeding cages to the presence of a litter of pups in either Type II (Welch’s *t*-test; *t*_24.3_ = −1.78, *P* = 0.09) or Type III (*t*_5.2_ = 0.17, *P* = 0.87) cages (Extended Data Fig. 2). The ammonia levels were comparable, regardless of whether a dam had given birth or not. For breeding pairs, we could not test for whether a litter of pups seemed to increase ammonia levels as we had only one breeding pair in a Type III cage and only one Type II cage was registered to have a litter of pups. We could not find any data supporting an effect of sex on ammonia build-up (Extended Data Fig. 3). Analyzing Type II cages in a two-way ANOVA using cage occupancy as a predictor, we could find no effect of sex on the intra-cage ammonia levels (F_1,392_ = 0.56, *P* = 0.45).

**Fig. 2:**
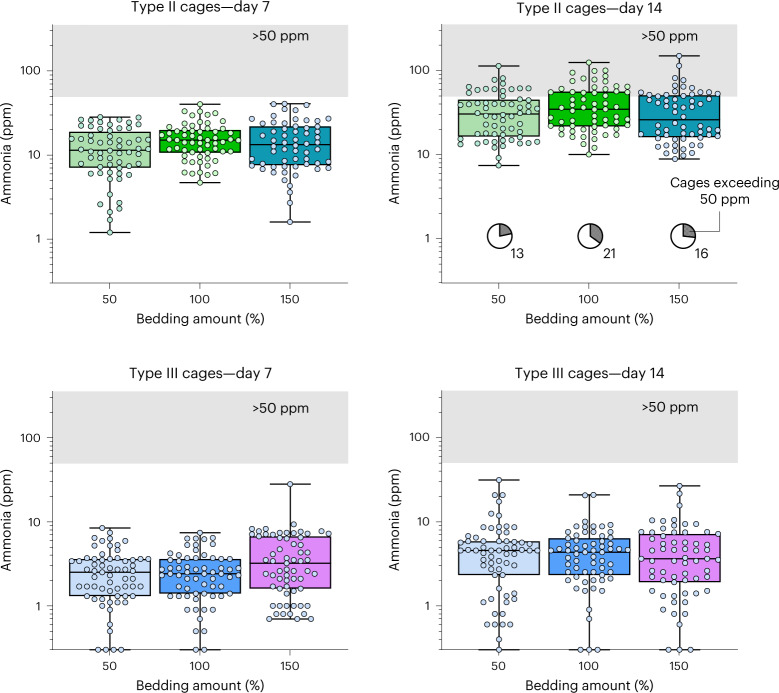
Varying the amount of bedding. Intra-cage ammonia is shown arranged by cage size (Type II (upper) and Type III (lower) cages) and amount of bedding (halving and doubling the standard volume of bedding, listed here as 100%). Intra-cage ammonia was sampled at day 7 (left) and day 14 (right) after cage change. Note that data are shown on a logarithmic axis and that the axis has been truncated at 0.3 ppm (censoring data between 0.1 and 0.3 ppm). Boxes display medians and the inter-quartile range, and whiskers denote full range of the data. The gray area is used to highlight the area of readings exceeding 50 ppm NH_3_. The number and proportion of Type II cages exceeding the 50 ppm threshold at day 14 is indicated at the bottom of the graph. For the exact composition of groups, see Table 2.

**Extended Data Fig. 4:**
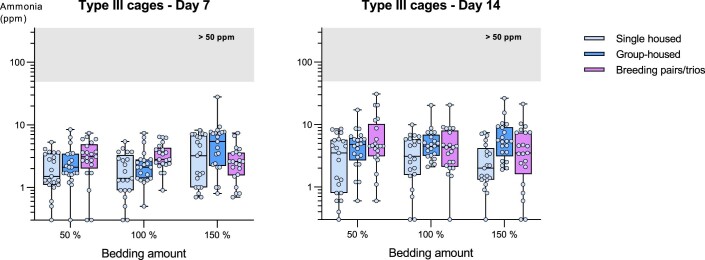
Ammonia levels with different amounts of bedding in Type III cages, separated by cage use. The Type III cages in Experiment 2 (Fig. 2) have been divided by the type of housing the cage was used for (see Table 2). There are no obvious trends outside a slightly lower ammonia build-up in cages with single-housed mice (F_2,60_ = 10.7, *P* < 0.001; Tukey’s post hoc test: *P* < 0.01 relative to both other groups). This factor did however not interact with the bedding amount. Boxes display medians and the inter-quartile ranges, whiskers denote full range of the data.

We also did not find an effect when measuring ammonia levels in cages with different amounts of bedding (Fig. 2). Given that three levels of bedding were assessed in all of the cages (Table 2), and that sampling was done at both 7 and 14 days, we would have expected our experiment to pick up any changes that we would have considered to be of a meaningful magnitude. Yet, no effect was found for either cage type (repeated-measures ANOVA using day-14 data, effect of bedding; *F*_2,242_ = 1.45, *P* = 0.24; cage × bedding interaction: *F*_2,242_ = 0.96, *P* = 0.39). Furthermore, splitting up the Type III cages by housing type (single housed, group housed; Table 2) did not reveal any hidden effects (Extended Data Fig. 4). There were significantly lower ammonia levels in cages of single-housed mice on day 14 (repeated-measures ANOVA, between-subjects effects; *F*_2,60_ = 10.7, *P* < 0.001; Tukey’s post hoc test: *P* < 0.01 relative to group-housed mice and breeding cages), but this effect did not interact with the bedding amount.

**Table 2:** Cages sampled with varying volumes of bedding

	Single-housed mice	Group-housed (2–7) mice	Breeding pairs/trios
**Smaller (Type II) cages**			
Number of cages (*n*)	–	–	60
Floor space per animal (cm^2^)	501	–	167–251
**Larger (Type III) cages**			
Number of cages (*n*)	21	21	21
Floor space per animal (cm^2^)	820	117–410	273–410

Note that each cage acts as its own control – sampled at 50%, 100% and 150% of the standard bedding amount (1.5 liters for Type II cages and 3.0 liters for Type III cages).

## Discussion

In the present study, we analyzed whether intra-cage ammonia levels could be brought down by changing the bedding volume. This was not the case. Whereas it is well established that using too little bedding in a cage will lead to a rapid increase in ammonia levels^34^, cages with a halved volume of bedding showed no signs of a change for the worse. Anecdotally, using too much bedding may lead to elevated ammonia levels in IVCs. The theory, related to us by cage manufacturers, is that a large bedding volume allows urine to seep to the bottom of the cage, where wet pockets can form, away from the drying effects of the circulated air in the cage. This ‘Goldilocks theory’ postulates that there is an optimal bedding amount. Using too much or too little may cause issues. We did not see any evidence of such an effect, however, when varying the amount of bedding within reason (0.5–1.5 liters in Type II cages; 1–3 liters in Type III cages). Consequently, we have to conclude that the ranges of bedding in our investigations were optimal, or near optimal.

A fair number of investigations have been made to identify bedding material that is highly absorbent and effective at reducing ammonia build-up^35–41^. Generally, wood chips (as used in the present study) and corncob have been shown to be appropriate for reducing ammonia build-up^22^. Although corncob has been shown to be slightly more absorbent than aspen wood chips, we are not convinced that the use of corncob could be useful in our present scenario (corncob is only more absorbent per volume of bedding; this effect is reversed if expressed per weight^37^). Moreover, rodents prefer cages lined with wood chips, over corncob, as shown in preference studies with mice^42,43^ and rats^44^, possibly owing to the denser, jagged, corncob ‘crumbs’ making for an uncomfortable surface to rest on^45^. Instead, we would like to draw attention to the volume of air in the cages.

In the present study, we found that our smaller (Type II) IVCs consistently accumulated higher intra-cage ammonia levels than did the larger (Type III) IVCs. Notably, this was the case also when mice were housed at comparable stocking densities in the two cage types. Even if an individual mouse was allotted the same amount of floor space, and consequently bedding, the smaller cages were more prone to accumulating ammonia. This observation suggests that cage volume, as opposed to living area, is of importance. Moreover, breeding trios appeared to generate more ammonia than did non-breeding groups of three. We could not find increased ammonia levels in the presence of a litter of pups. Instead, a behavioral component, inherent to breeding animals, may influence ammonia build-up (as reported before^31^). Perhaps the increased water and feed consumption by pregnant females can partly explain the higher ammonia concentrations found in breeding cages.

Whereas it has been suggested that male mice produce more ammonia than females (whether simply by being larger and having more ‘biomass’^46,47^ or by drinking more water and consequently generating more urine^48^), we have repeatedly been unable to substantiate such an effect. Under the conditions of our investigation, males and females housed at comparable densities appeared to produce comparable ammonia levels (Type II cages, Extended Data Fig. 3). If there is an effect of sex, this was overshadowed by other parameters. We were unfortunately not able to do a meaningful comparison of strains.

The airflow in IVCs is designed, and tested, using empty cages. This practice is understandable, given that a manufacturer cannot predict the type or amount of bedding, enrichment items, shelters or nest building materials that will be used in any one particular animal house. Yet, these additional items are required by European legislation^49^ and strongly recommended in North American guidelines^50^. In smaller-volume cages, with less headspace, items such as shelters and nest-building material may divert the airflow considerably. We suspect this may be the case for our smaller (Type II) cages. Whereas, for example, breeding pairs/trios housed in these smaller cages live up to EU requirements regarding cage space (defined as floor space), it is clear that these housing conditions create an undesirable environment. We will note that different models of IVCs employ different directions/patterns of airflow and different rates of flow. Consequently, we cannot know to which degree cage provisioning affects aeration across different models; we can however strongly recommend investigating this.

We set the maximum housing density in our cages by calculating the floor space available to a single mouse—essentially treating floor space as a consumable resource. Doing the same for the volume of air in the cages may be less sensible. In our experiments, the larger cages had an approximate volume of 15 liters, ignoring volume displaced by feed, bedding, nesting material and the animals themselves. The smaller cages measured approximately 9 liters (experiment 1) and 8 liters (experiment 2). The difference in volume between the cage types is not greatly different from the difference in floor space. Instead, we believe that a certain total headspace may be needed in a cage for appropriate aeration with respect to avoiding ammonia build-up. The volume of a cage and the number of mice that can be housed in it without risking high levels of intra-cage ammonia may thus not follow a simple relationship. Optimal housing conditions may not be easily predicted, but rather have to be established through practical experiments. Factors like geometry of the cage, airflow pattern, cage provisioning (shelters, nesting material and enrichment items) are just a few factors that may have an important influence on ammonia levels.

In the present investigation we were unable to control for factors such as age, weight or genetic background of the mice in the studied cages. We could also not manipulate the cage environment beyond changing the bedding amount within reason. Whereas this provided us with an appropriate overview of the situation in our facilities, the data cannot answer questions with regard to the influence of other parameters that may have an influence on ammonia build-up. Mice’s ability to maintain a consistent latrine location was shown to greatly influence intra-cage ammonia levels in a recent study^46^. Encouraging mice to place their latrine location in the front of the cage—the area that was associated with the lowest ammonia build-up—could offer a simple behavioral means for reducing cage ammonia levels that is worth investigating. In follow-up investigations, we also hope to be able to answer whether other elements—amount and types of cage furniture and placement of the food hopper—might further influence intra-cage ammonia levels.

With new designs being introduced for mouse IVCs, where cages are made even more compact by lowering the ceiling of the cages, we may see even greater issues with ammonia build-up. A worsening factor is that this problem is not easily detected because animal caretakers and research personnel are not breathing the same air as the one circulated in the cages. We were clueless of our ammonia issues before we started sampling cages. In the present report, we present the levels measured at the very end of a cage change cycle. It can be argued, consequently, that they paint a worst-case scenario. The mice are only exposed to these levels toward the very end of a 14-day period. Yet, we would not like mice to be exposed to ammonia levels exceeding 50 ppm at any point in our facilities. In fact, we would optimally like to maintain ‘human-friendly’ levels below 25 ppm throughout. Whereas this could fairly easily be addressed by adopting a shorter cage change cycle, this would not be without consequences. It is well established that mice find constant handling and reordering of their home environment stressful^51^; it disrupts their behavioral patterns and circadian rhythm^52,53^, and cage cleaning (removing odor cues) may provoke aggression in male mice^54,55^. More frequent cage changes are not only more laborious on the part of the animal caretakers, but also disliked by the cages’ inhabitants. Guided by our data, we instead tentatively suggest that, when allotting living space to our mice, we keep in mind also the volume of the cages and not just the floor space.

## Methods

All mice were housed in IVCs (Tecniplast) of either a Euro Standard Type II (experiment 1: ‘1285L’, 365 × 207 × 140 mm; experiment 2: ‘GM500’, 391 × 199 × 160 mm) or Type III (‘1290D’, 425 × 276 × 153 mm) design. The animals were housed on aspen wood chips (Tapvei), kept on a 12:12 hour light–dark cycle, with 30 min of twilight at transitions. Ambient temperature was maintained at 19–23 °C and humidity at 42–46%, and cages were ventilated at a rate of 65–75 h^−1^ air changes under negative pressure. The cage changing frequency was once every 2 weeks throughout. Animals were provided shelters—red semi-transparent plastic shelters (‘JAKO’; Molytex), and cardboard tubes (Lillico)—nesting material (‘Happi-mat’ nestlets; Scanbur) and gnawing sticks (Tapvei). Extruded feed (‘Altromin 1314’, Brogaarden) and tap water (provided in bottles) were provided ad libitum. The sampled cages were all situated in barrier breeding units that are continuously tested for pathogens in line with the Federation of European Laboratory Animal Science Associations’ recommendations^56^. The animals were all considered specific pathogen free.

Intra-cage ammonia was measured with a handheld photoionization detection sensor (Tiger LT detector; Ionscience), similarly to previous studies^34,57^. No other organic compounds that could potentially interfere with the measurements were expected to have been present at any appreciable concentration in any of the cages. The measured concentrations have been reproduced using electro-chemical measurements to ensure the accuracy of the photoionization detection measurements. A crude probe was fashioned out of plastic tubing that allowed sampling through the water bottle port (similarly to other studies^22^), while cages were still on the rack. The cages were consequently (mostly) undisturbed, and actively ventilated, while sampling. Readings were obtained from the center of the cage, approximately 5 cm above the bedding. The lower limit of detection of the instrument was 0.1 ppm ammonia. The readings were obtained live, precluding blinding of the samplings.

All of the data in the study were obtained through opportunistic sampling of animals engaged in other experiments or breeding programs. With no previously agreed-upon depth of bedding for cages in the University’s animal care and use program, changes in bedding volumes were made within what was considered to be the normal range for standard housing. These changes were made as a part of the facility’s continuous improvement work. Cage manufacturers had suggested that a cause of the elevated ammonia levels could be the use of too much bedding substrate, prompting investigation. All of the procedures were carried out under supervision of the local animal welfare committee and the program’s clinical veterinarians.

Hypothesis testing was carried out on log-transformed data (*X*′ = log_10_(*X* + 1), to account for zero values) throughout, since data appeared to be (roughly) log-normally distributed. Welch’s *t*-test was used for simple comparisons, whereas ANOVA models were used when there was more than one independent variable. For post hoc comparison of multiple groups, we employed Tukey’s (HSD) test. To compare animals housed at similar densities across the two cage types, we chose to focus on cages with mice housed at a density of 140 cm^2^ per animal or less. This produced similar ranges of 88–133 cm^2^ per animal for Type II cages and 91–137 cm^2^ per animal for Type III cages with large enough sample sizes for statistical testing. With the same cages being used for the three levels of bedding, we were able to employ a repeated-measures ANOVA. Linear regressions were carried out to scrutinize the relation between housing density and ammonia build-up. Best-fit slopes were then tested using ANOVA. Analyses were carried out in SPSS v.28 (IBM), throughout the analyses two-sided testing was used and *P* < 0.05 was considered significant.

### Experiment 1

To verify our previous in-house findings, randomly chosen IVCs were sampled across two animal housing units (Table 1). A total of 451 unique Type II cages and 73 unique Type III cages housing varying numbers of animals were sampled 10–14 days after cage change.

### Experiment 2

In the second experiment (Table 2), changes were made to the routine housing conditions for cages used for breeding. Cages were provided the standard amount of bedding utilized in the animal facilities (Type II cages: 1.0 liter; Type III cages: 2.0 liters), 50% of the standard amount (Type II cages: 0.5 liters; Type III cages: 1.0 liter), or 150% of the standard amount (Type II cages: 1.5 liters; Type III cages: 3.0 liters). A full cross-over design was employed, where each cage was provided with each level of bedding, in a random order. The design was a balanced Latin square with equal numbers of cages for each condition in each time block. Cages fell into one of three categories—breeding pairs (one male, one female) or trios (one male, two females), single-housed males (currently not used for breeding) or group-housed mice (weaned litters of young mice).

### Reporting summary

Further information on research design is available in the Nature Portfolio Reporting Summary linked to this article.

## Online content

Any methods, additional references, Nature Portfolio reporting summaries, source data, extended data, supplementary information, acknowledgements, peer review information; details of author contributions and competing interests; and statements of data and code availability are available at 10.1038/s41684-023-01179-0.

## Supplementary information

Reporting Summary

## Data Availability

All of the raw data have been made available online (10.6084/m9.figshare.21829353).
